# 3D Slicer open-source software plug-in for vector-based angle calculation of canine hind limb alignment in computed tomographic images

**DOI:** 10.1371/journal.pone.0283823

**Published:** 2024-03-29

**Authors:** Juliette Burg-Personnaz, Martin Zöllner, Sven Reese, Andrea Meyer-Lindenberg, Andreas Brühschwein

**Affiliations:** 1 Centre of Veterinary Clinical Medicine, Clinic of Small Animal Surgery and Reproduction, Veterinary Faculty, LMU, Munich, Germany; 2 Department of Veterinary Sciences, Veterinary Faculty, Institute of Veterinary Anatomy, Histology and Embryology, LMU, Munich, Germany; Brunel University London, UNITED KINGDOM

## Abstract

**Background:**

Severe and complex angular limb deformities in dogs require accurate morphological assessment using diagnostic imaging to achieve successful orthopedic surgery. Computed tomography (CT) is commonly used to overcome projection errors in two-dimensional angular measurements of dog hindlimb alignment. Three-dimensional volume rendering (VR) techniques permit virtual positioning and variable projection, but the final CT-image that defines the projection plane for angular measurements remains two-dimensional.

**Objective:**

We wanted to develop a true three-dimensional open-source technique to measure the alignments of the hind limbs of dogs in CT scanners.

**Methods:**

We developed an open-source 3D Slicer plug-in, to perform angular measurements using vector calculations in three-dimensional space. In 113 CT-scans of canine pelvic limbs, femoral torsion, femoral varus, femorotibial rotation, tibial torsion, tibial varus and tibiotalar rotation angles were calculated and compared to an already validated technique using VoXim®.

**Results:**

Reference points were identified and measurements were possible in the 113 acquisitions. The greatest difference between the two techniques was 1.4° at only one tibial torsion angle. Mean values for all Bland-Altman plots did not show significant differences and were less than 0.07° for all comparisons.

**Discussion:**

Based on these results we considered angular measurements of canine hind limb alignment in CT scans using the 3D Slicer extension program sufficiently accurate for clinical orthopedic and surgical purposes in veterinary medicine.

**Conclusion:**

With our open-source 3D Slicer extension software, we provide a free accessible tool for veterinary orthopedic surgeons and thus we hope to improve angular measurements in CT-scans of canine hind limb deformities through true three-dimensionality.

## Introduction

Angular deformities of the hind limbs are common in dogs with patellar dislocation [1–14], posttraumatic bone deformities caused by fracture malunions [15–18] and abnormal physeal growth due to partial or complete premature physeal closure [19, 20]. These may result from trauma [18], genetic factors in the chondrodystrophic dachshund [21, 22] and other musculoskeletal genetic diseases [23]. Most cases of patellar dislocation are thought to be of developmental origin [24, 25] and are considered to be a complex skeletal malformation, affecting the entire hind limb alignment [24, 25]. Abnormal femoral neck inclination (coxa vara and valga) and version angles as well as abnormal femoral and tibial torsion, varus and valgus angles might be associated with patellar dislocation [24, 25]. Therefore, dogs with patellar luxation may benefit from additional corrective osteotomies [3, 5, 8, 10, 11, 18, 26]. Severe and complex angular hind limb deformities in dogs require accurate morphological assessment using diagnostic imaging to achieve successful orthopedic surgery [15, 16, 27]. Angle measurements are commonly performed using radiography to determine osseous deformities in the canine femur [3, 5, 8, 10, 11, 18] and tibia [19–21, 28, 29]. In these radiographs, a transformation and reduction of a three-dimensional object into a two-dimensional image occurs [30]. Therefore, the landmarks used to create lines which are defining the angles are always in the same plane. The summation of structures along the trajectory of the divergent X-ray beam leads to superimposition, magnification and distortion in two-dimensional radiographs [30]. The measurement error caused by these projection effects is counteracted by standardization of positioning as well as beam centering and alignment. A variation in positioning can affect angle measurements, which has been demonstrated for distal femoral varus angles [31–35]. Bone deformities are particularly difficult to evaluate radiographically [28, 36, 37], specifically if deformities are present in more than one plane as described for the antebrachial torsion which interferes with the measurement of varus/valgus [38]. Computed tomography (CT) is commonly used to overcome two-dimensional projection errors [2, 4, 6, 7, 9, 12, 16, 17, 27, 32, 39–48]. Although CT generates true three-dimensional data, most CT measurement techniques are still two-dimensional. Measurement points and their axes from different CT-images are commonly transferred into one measurement plane, using a summation of two individual transverse CT-images into one single superimposed image [28, 43, 46]. Image processing with the help of specific software using multiplanar reconstruction (MPR) [4, 39–41, 49], maximum intensity projections (MIP) [39] and volume rendering technique (VR) [9, 15–17, 27, 32, 39, 40, 42, 44–46, 48, 50, 51] allow the generation of nonaxial two-dimensional images and virtual radiographic positioning, but it results into the creation of a planar image that lacks a third dimension. Anatomical landmarks used for angle measurement are often not in one plane and a compromise must be made, usually by using a standardized radiographic projection selected by the operator’s visual judgement. Variations in these virtual three-dimensional views and projections limit the measurement of complex combined angles and torsional deformities [16].

CT-scanners create, and CT-data contain true three-dimensional information based on three-dimensional Cartesian coordinate systems and the standard Digital Imaging and Communication in Medicine (DICOM) regulates the technical details [52, 53]. Three-dimensional information and Cartesian coordinate systems allow three-dimensional vector calculation that can be used to measure angles three-dimensionally [54, 55]. The mathematical definition of a vector from point A to point B is the coordinate-wise difference B-A [54]. The smallest angle θ between two vectors $\vec{a},\vec{b}$can be calculated using the scalar-product $\vec{a}\cdot \vec{b}=x_{a}x_{b}+y_{a}y_{b}+z_{a}z_{b}=\|\vec{a}\|\|\vec{b}\|\cos\;\theta$. This angle is measured in the plane defined by the normal vector [54]. If the three-dimensional coordinate system is changed, the angle between the two vectors remains the same, so the vector calculation itself is independent of the reference frame and the three-dimensional coordinate system used [55].

As reported in the open-source software 3D Slicer [56, 57], a scanner-independent, patient-centered physical space coordinate frame reference systems allow for coherent integration and visualization of multiple image and data types in three dimensions. Having truly three-dimensional information based on CT-data and three-dimensional open-source tools to calculate angles three-dimensionally using vector calculations, we are able to standardize the projection planes for angular measurements using three-dimensional mathematical definition, rather than using visual judgment only.

The goal of this study was to develop an open-source technology to measure canine hind limb alignment in a truly three-dimensional fashion, including femoral torsion, femoral varus (or valgus), femorotibial rotation, tibial torsion, tibial varus (or valgus) and tibiotalar rotation angles.

## Material and methods

### Software

**Fig 1:**
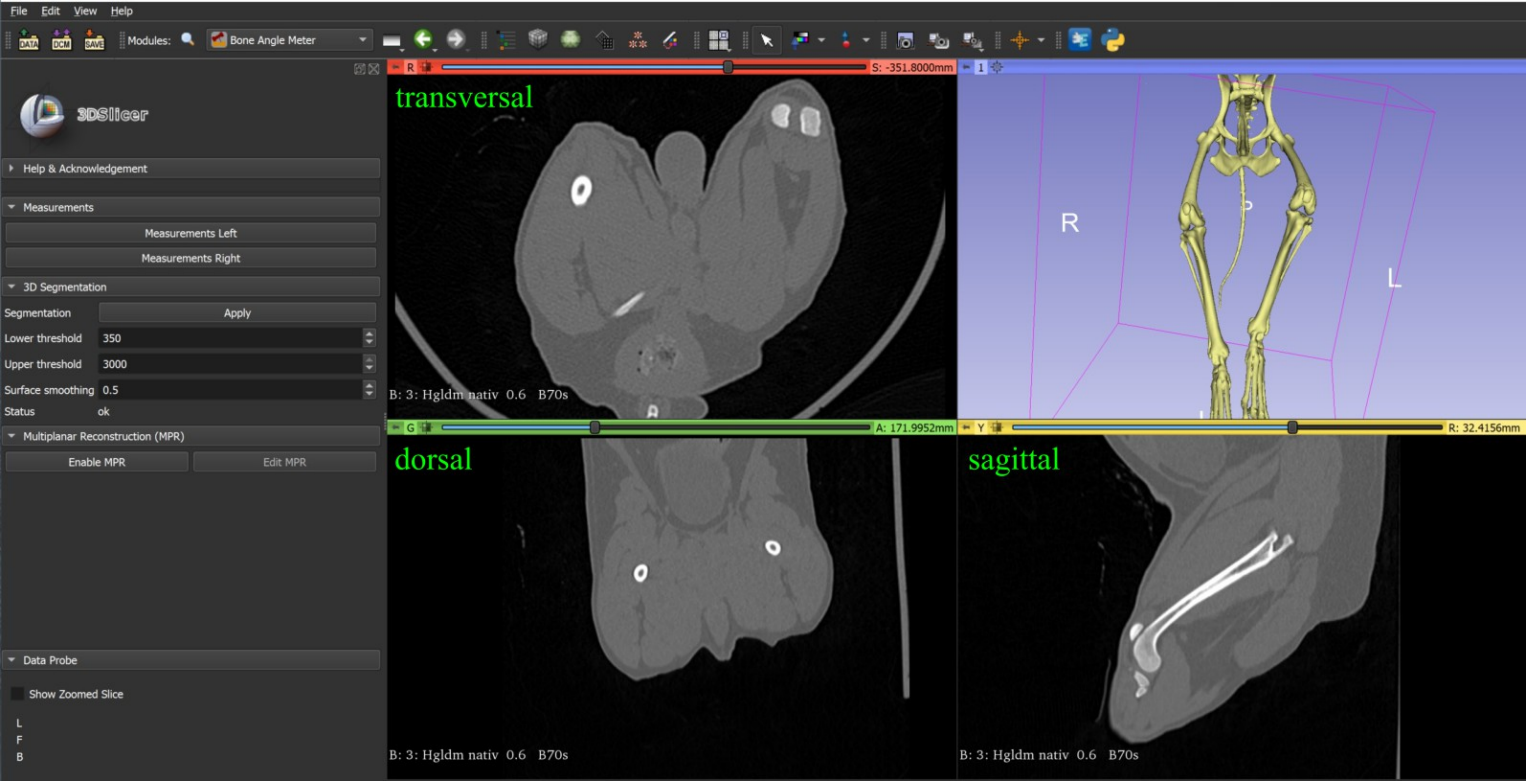
3D Slicer view of the programmed plug-in.

The clinical research tool 3D Slicer (Version 4.11.20210226, www.slicer.org), a free open-source desktop software application and development platform for medical and biomedical imaging research designed for multi-modality three-dimensional applications based on a three-dimensional RAS (Right, Anterior, Superior) patient Cartesian coordinate system, was used for this project [56, 57]. 3D Slicer is not tied to a specific hardware, but otherwise similar to a radiology workstation, supports versatile visualizations and is designed to facilitate the development of new functionality. New tools and features can be programmed in the form of plug-ins and added to the main software by additional installation in the form of 3D Slicer extensions, which can be redistributed [56, 57]. We developed and programmed an open-source plug-in for the 3D Slicer software [56, 57] to view and morphologically analyze CT-data with the goal of three-dimensional angular measurements. Therefore, we used a three-dimensional coordinate system and vector calculations triggered by manually set reference points in the transverse, sagittal, dorsal plane and a three-dimensional volume-rendered model of the virtual reconstructed skeleton (Fig 1).

### Mathematical method of angular measurements

#### Vector based definition of projection planes

To standardize angular measurements, we mathematically defined the projection planes in which the angle measurements between two projected vectors take place. To determine the bone’s torsion angle, we used a projection plane that was orthogonal to the longitudinal axis of the bone. The longitudinal axis was represented by the vector $\vec{n_{P}}$ perpendicular to the projection plane. To determine a medial or lateral deviation of the longitudinal bone axis, which is equivalent to a varus or valgus angle, we required a mathematical definition of a dorsal projection plane. To define the dorsal projection plane, we calculated its normal vector $\vec{n_{P}}$, based on the fact that two non-parallel vectors $\vec{p_{1}}$ and $\vec{p_{2}}$ can define a plane and we calculated the normal vector $\vec{n_{P}}$ using the cross-product: $\vec{n_{P}}=\vec{p_{1}}\times \vec{p_{2}}$. As defined by the cross-product, the vector $\vec{n_{P}}$ is perpendicular to both vectors $\vec{p_{1}}$ and $\vec{p_{2}}$ and is therefore a normal vector to the plane *P*.

#### Vector based angular measurements

To measure angles with individual reference points and their three-dimensional coordinates in a three-dimensional CT coordinate system, we used the following mathematical vector calculations and projections. Any vector $\vec{a}$ can be written as $\vec{a}={\vec{a}}_{∥}+{\vec{a}}_{⊥},$ where ${\vec{a}}_{∥}$ is a vector lying in the plane and ${\vec{a}}_{⊥}$ is a vector perpendicular to the plane, i.e. parallel to the normal vector ${\vec{n}}_{P}$. We were interested to find ${\vec{a}}_{∥}$, which is the projection of $\vec{a}$ to *P*. Hence, we restore the vertical component first by

$$\vec{a}\cdot \vec{n_{P}}=({\vec{a}}_{∥}+{\vec{a}}_{⊥})\cdot \vec{n_{P}}=\underset{0}{\underset{︸}{{\vec{a}}_{∥}\cdot \vec{n_{P}}}}+{\vec{a}}_{⊥}\cdot \vec{n_{P}}=\|{\vec{a}}_{⊥}\|\|\vec{n_{P}}\|\Rightarrow \|{\vec{a}}_{⊥}\|=\frac{\vec{a}\cdot \vec{n_{P}}}{\left\|\vec{n_{P}}\right\|}.$$

We used the fact that ${\vec{a}}_{∥}$ and $\vec{n_{P}}$ are perpendicular and thus had a scalar product of 0. In the last step, we followed the fact that ${\vec{a}}_{⊥}$ and $\vec{n_{P}}$ are parallel which means that cos *θ* = 1. Therefore, we can calculate ${\vec{a}}_{⊥}=\|{\vec{a}}_{⊥}\|\frac{\vec{n_{P}}}{\left\|\vec{n_{P}}\right\|}=\frac{\vec{a}\cdot \vec{n_{P}}}{{\left\|\vec{n_{P}}\right\|}^{2}}\vec{n_{P}}$.

We found the projection ${\vec{a}}_{∥}$ as ${\vec{a}}_{∥}=\vec{a}-{\vec{a}}_{⊥}=\vec{a}-\|{\vec{a}}_{⊥}\|\frac{\vec{n_{P}}}{\left\|\vec{n_{P}}\right\|}=\vec{a}-\frac{\vec{a}\cdot \vec{n_{P}}}{{\left\|\vec{n_{P}}\right\|}^{2}}\vec{n_{P}}$.

Finally, to determine the angles projected into the desired plane we calculated the angle *θ*_*P*_ between the vectors $\vec{a}$ and $\vec{b}$ in plane *P* using the scalar product:

$$\theta_{P}=\cos^{-1}\left(\frac{{\vec{a}}_{∥}\cdot {\vec{b}}_{∥}}{{\|\vec{a}}_{∥}\|\|{\vec{b}}_{∥}\|}\right)$$

### Anatomical description of reference points, projection planes and angular measurements

#### Femoral antetorsion angle

**Fig 2:**
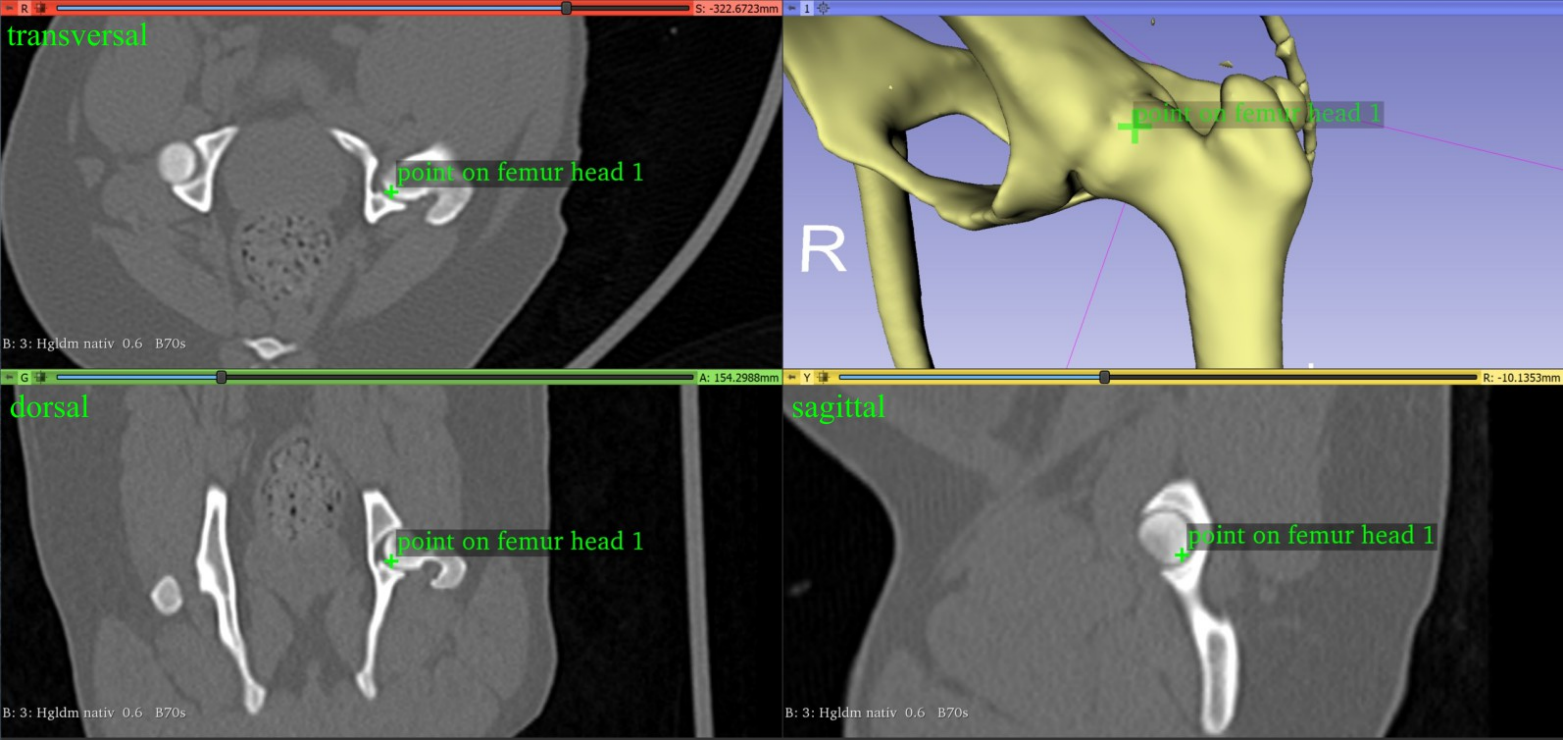
One reference point along the capital bearing area of the femoral head of the left femur of a dog to calculate the center of the femoral head.

**Fig 3:**
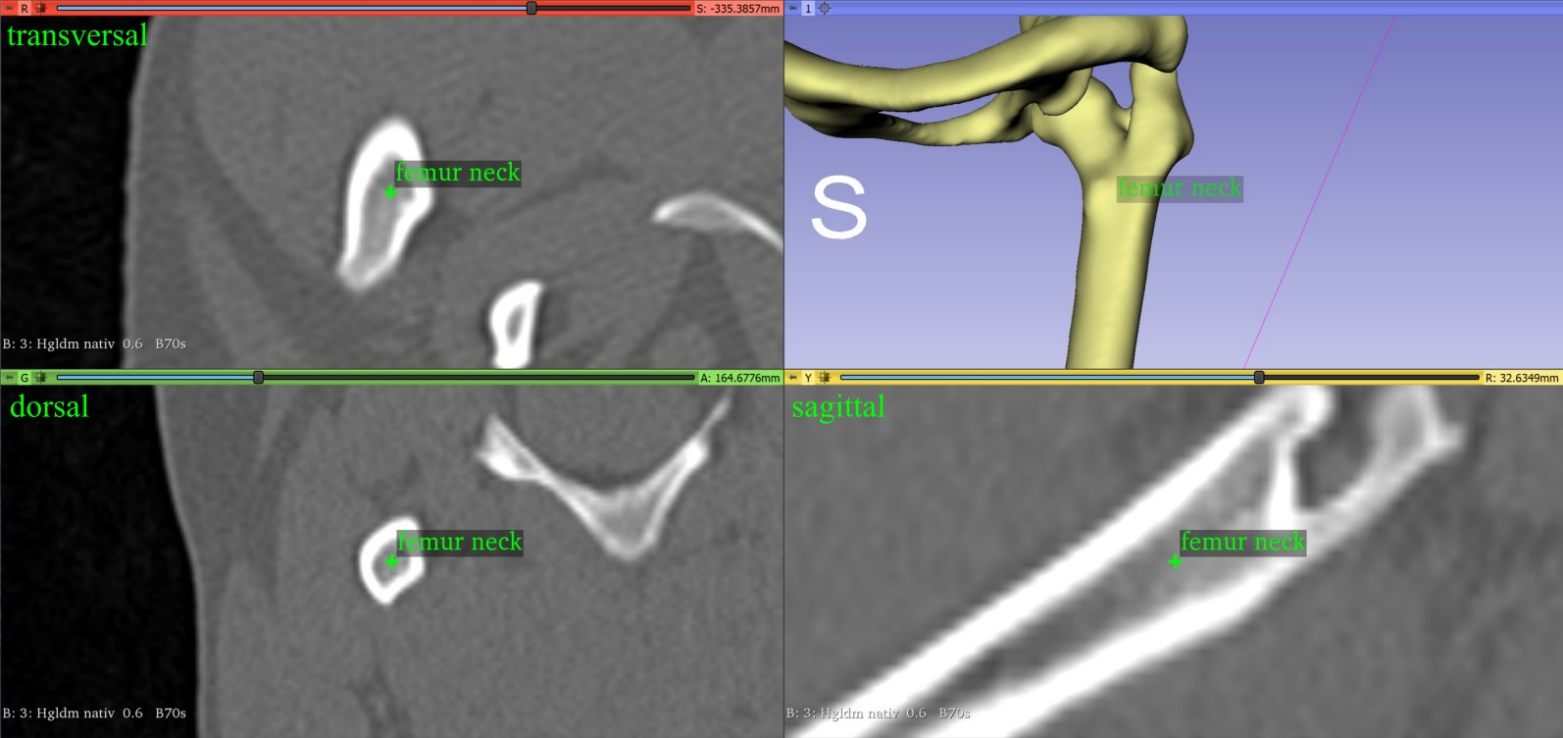
The femoral neck base center of the left femur of a dog manually placed in the center of the proximal femoral metaphysis, at the level of the highest medial protrusion of the lesser trochanter.

**Fig 4:**
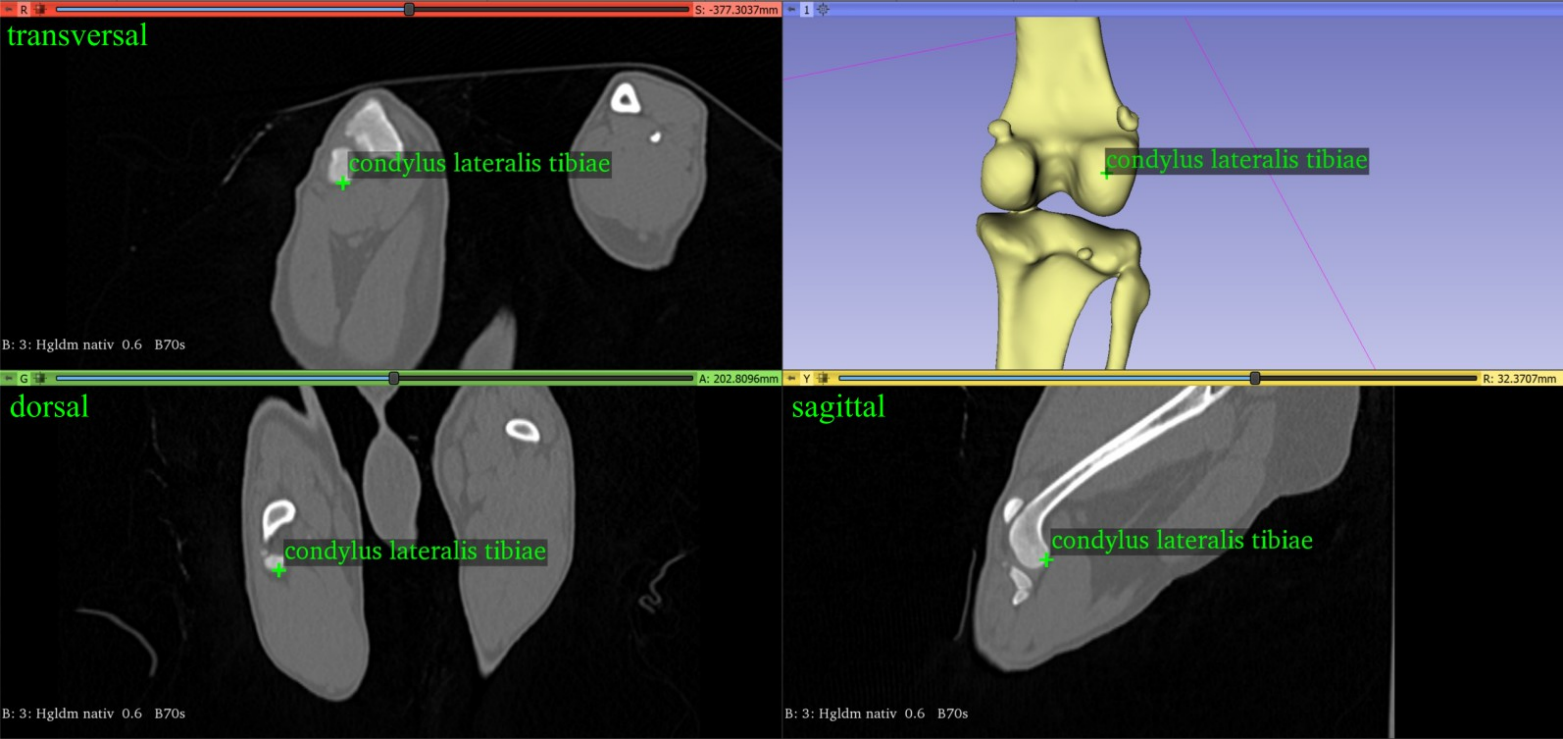
The most caudodistal midpoint on the convex surface of the lateral condyle of the right femur of a dog.

We calculated the antetorsion angle of the femur using the center of the femoral head, which we defined as the midpoint of a three-dimensional sphere. Reference points were set along the capital bearing area of the femoral head (Fig 2), and a least squares method was used to calculate the fitted sphere and its center [58]. The femoral neck base center was manually placed at the center of the proximal femoral metaphysis, at the level of the highest medial protrusion of the lesser trochanter [13, 14, 32] (Fig 3). Two points at the center of the lateral and medial condyles were manually set at the midpoint of the most caudal and distal points on the surface of the convex femoral condyle (Fig 4). We needed two points to define the projection plane based on the normal vector, and we used the proximal and distal femoral diaphyseal centers. As the center of a circle of a diaphyseal transverse bone cross-section, the proximal femoral diaphyseal center was set at the level of transition between the proximal, and middle thirds, and the distal femoral diaphyseal center between the middle and the distal third of the femoral diaphysis [32]. The antetorsion angle of the femur was calculated using two vectors: the distal femoral condyle line, which was the line between the center of the lateral and medial femoral condyle, and the femoral neck axis, which was defined as the line between the femoral head center and femoral neck base center. Both vectors were projected in the transverse plane defined by its normal vectors along the longitudinal axis of the femur between the distal femoral diaphyseal center and the proximal femoral diaphyseal center.

#### Femoral varus or valgus angle

We calculated the varus or valgus angle of the femur using two vectors: the femoral transcondylar axis, which was the line between the center of the lateral and medial femoral condyle [40], and the femoral longitudinal axis, which was defined as the line between the proximal and distal femoral diaphyseal center [32, 40]. Both vectors defined the dorsal femoral plane. Therefore, a projection was not necessary. We considered a “valgus” the lateral angular deviation of the femoral transcondylar axis when the angle was negative. The medial deviation of the distal femoral axis was considered a “varus” when the angle was positive.

#### Femorotibial joint rotation angle

**Fig 5:**
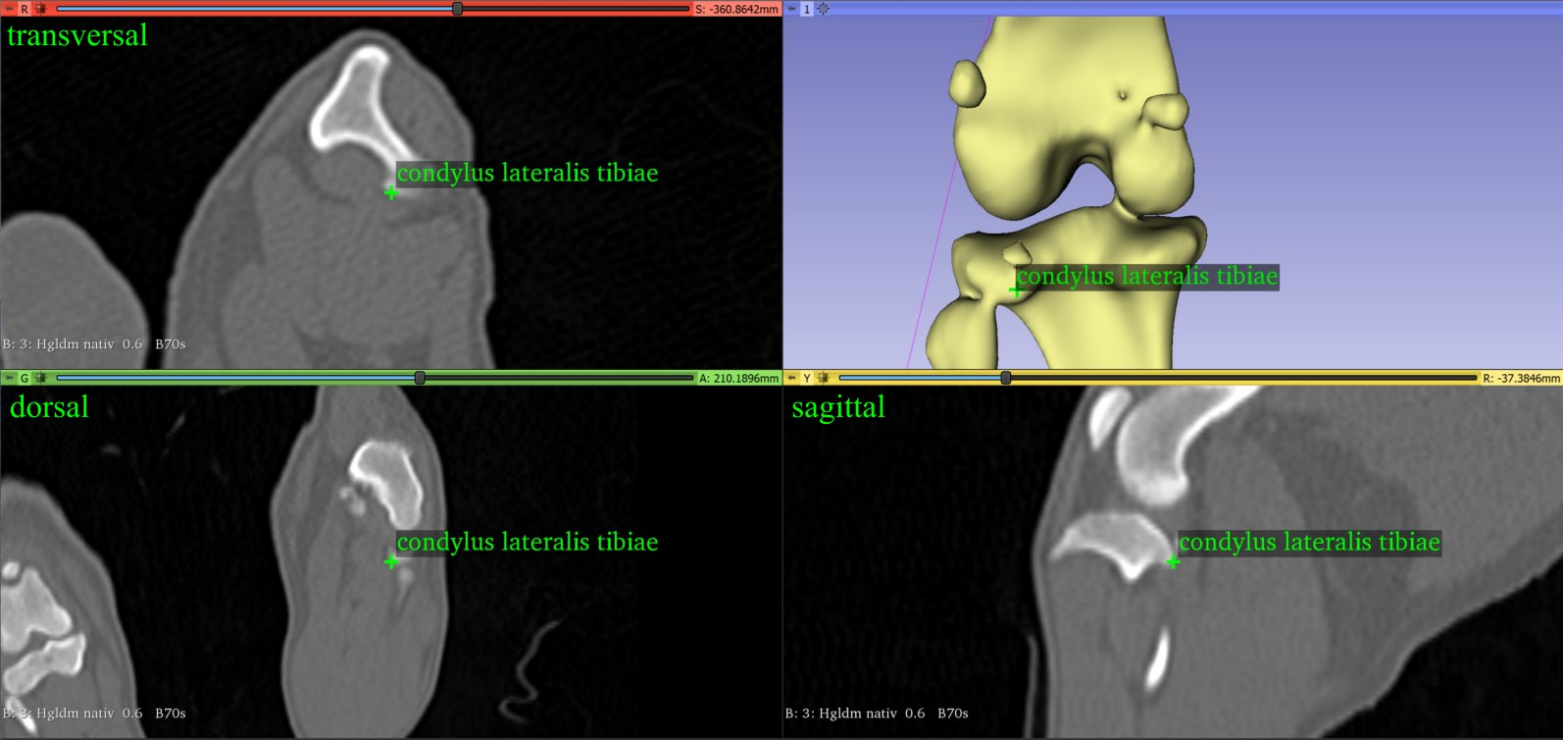
The most caudal point of the lateral condyle of the left tibia of a dog.

We needed two vectors to measure the femorotibial joint rotation angle: the femur transcondylar axis, as defined for the antetorsion angle of the femur, and the proximal tibial line, defined by the most caudal protuberance of the condylus medialis and lateralis tibiae (Fig 5) [13, 14].

Both vectors were projected into a transverse plane whose normal vector is the tibial longitudinal axis. The tibial longitudinal axis was the line between the midpoints of the proximal and the distal tibial shaft. The first one was set to the middle of the diaphysis at the height of the foramen nutricium and the second one was set to the middle of the diaphysis at the height of the distal third of the tibia shaft. If the proximal tibia was rotated laterally, the angle was defined as negative and we obtained an external rotation angle. If the proximal tibia was rotated medially, the angle was defined as positive and we obtained a medial rotation angle.

#### Tibial torsion angle

**Fig 6:**
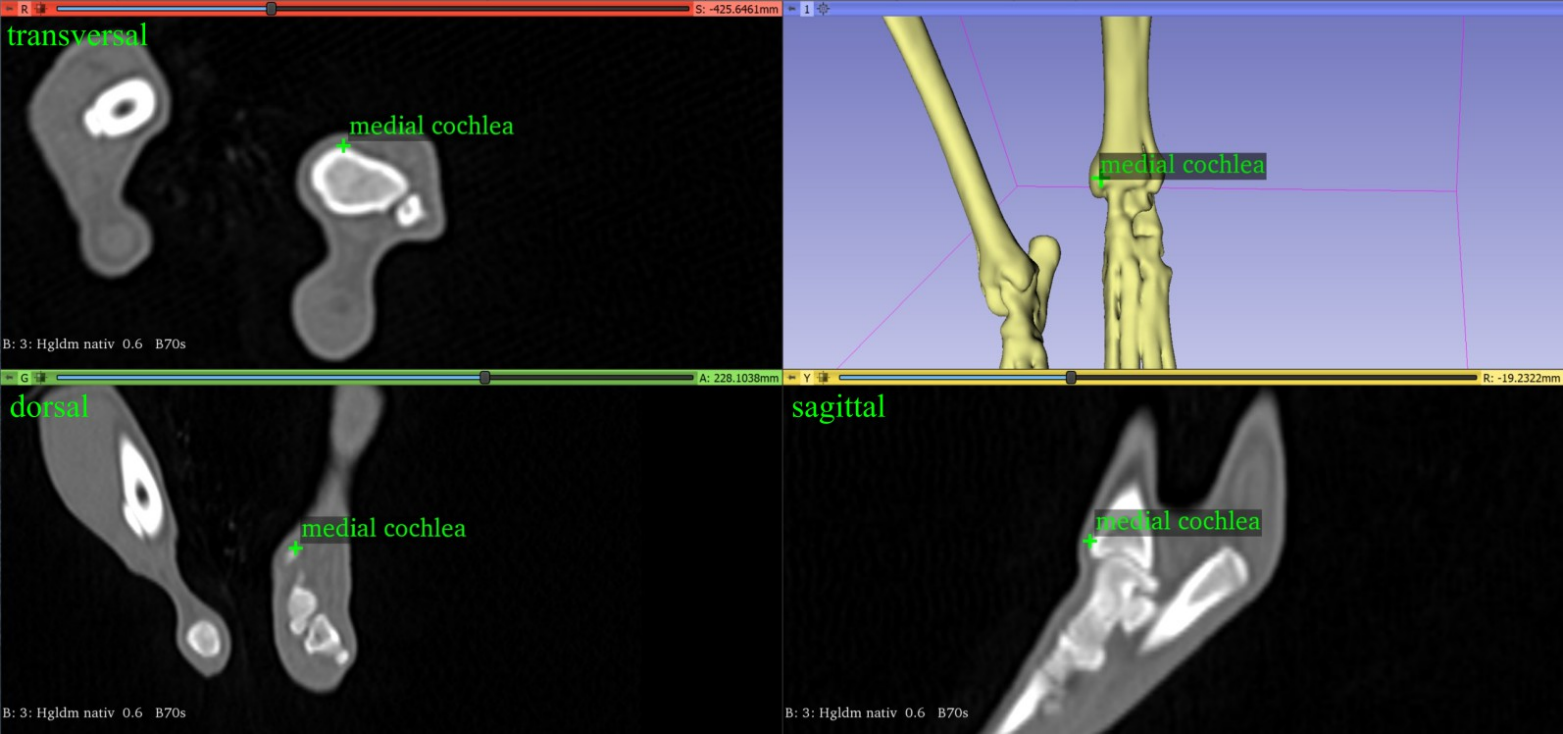
The most cranial point on the lateral part of the cochlea tibiae of the left tibia of a dog.

The tibial torsion angle was calculated using two vectors, the distal tibial front line and the proximal tibial caudal line. The distal tibia front line was defined as the line between the most cranial point lateral and medial on the cochlea tibiae [13, 14, 28] (Fig 6). The proximal tibial caudal line was the line between the most caudal protuberance of the medial and lateral condyle of the tibia [13, 14] (Fig 5). Both vectors were projected into a transverse plane defined by the tibial longitudinal axis as the normal vector. We defined a tibial torsion as an external torsion when the distal tibia line was rotated in the lateral direction to the proximal tibial line. In this case the angle was defined as negative. We defined a tibial torsion as an internal torsion when the distal tibial line was rotated in the medial direction to the proximal tibial line. In this case the angle was defined as positive.

#### Tibial varus or valgus angle

**Fig 7:**
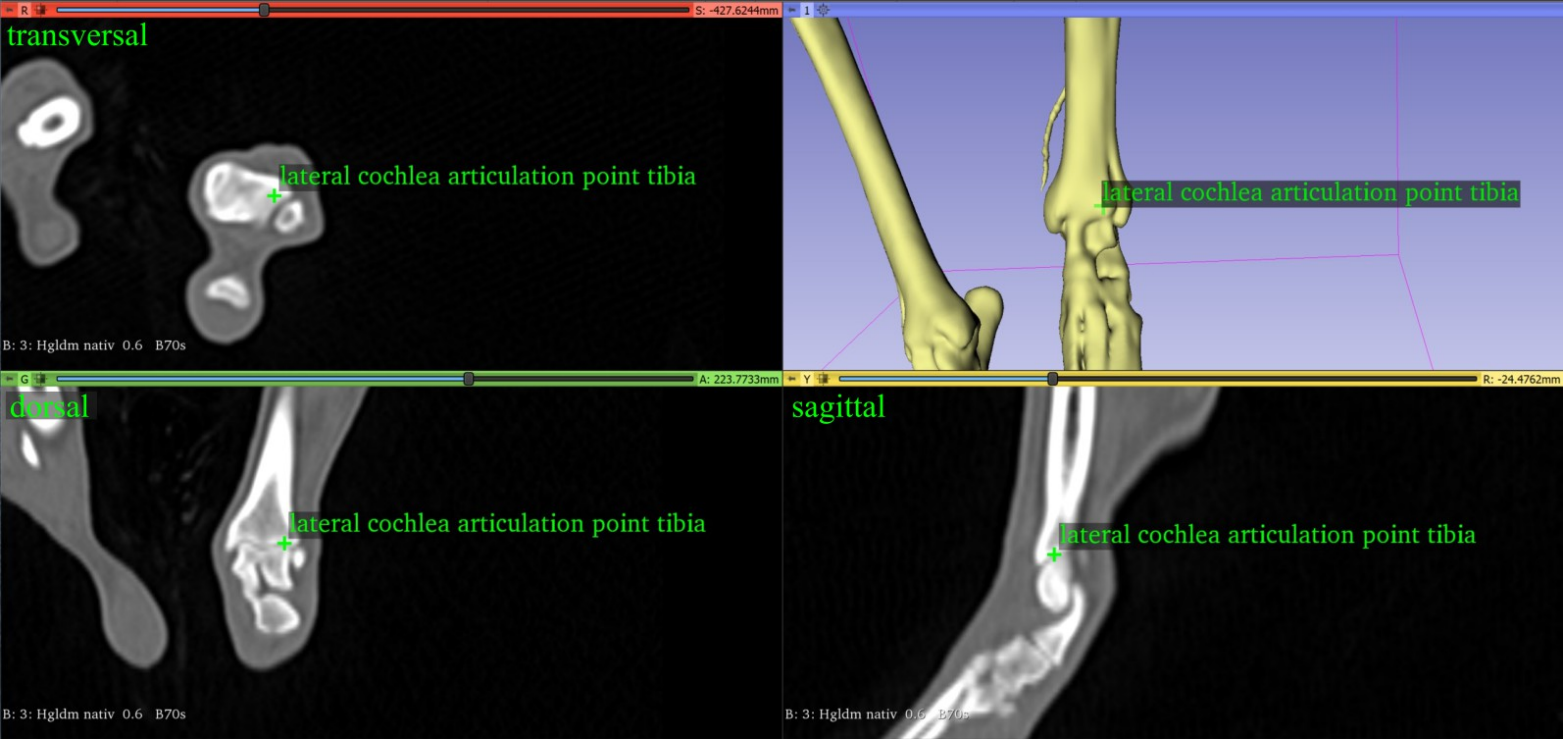
The lateral lowest midpoint of the cochlear-tibial groove of the left tibia of a dog.

**Fig 8:**
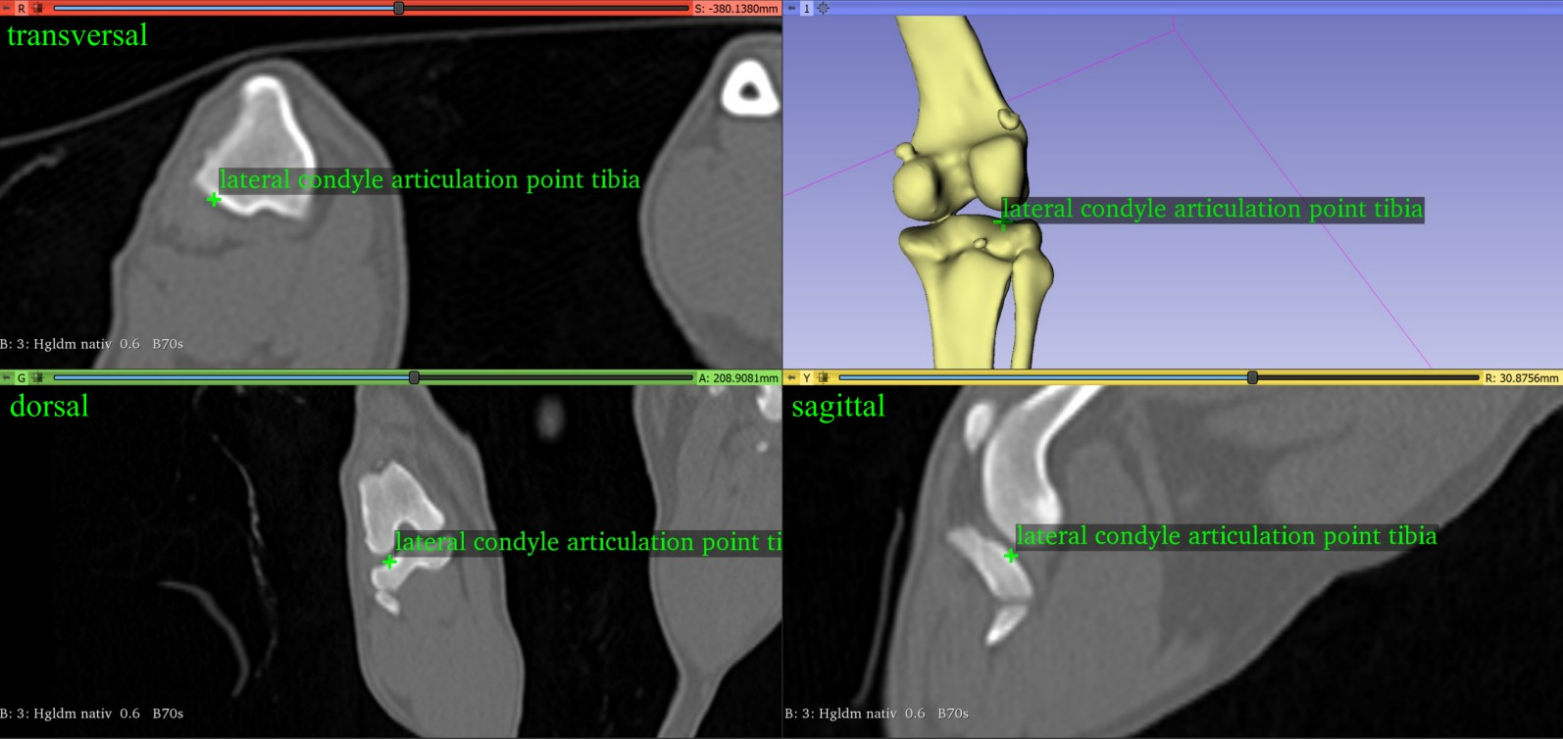
The lateral lowest midpoint of the condylar-tibial groove of the right tibia of a dog.

The angle between the distal tibial joint line and the proximal tibial joint line was termed tibial varus or valgus. The distal line was defined as the line between the lateral and medial deepest midpoint of the cochlear groove of the tibia (Fig 7). The proximal tibial joint line was defined as the line between the lowest midpoint of the lateral and medial articular groove of the tibial condyle at the center of the lateral and medial tibial plateau (Fig 8). Both, the distal tibial joint line and the proximal tibial joint line were projected onto the dorsal tibial plane defined by two vectors, the tibial longitudinal axis and the proximal tibial line. The angle lateral deviation of the distal tibial articulation line was considered a “valgus” if the angle was positive. The medial deviation of the distal tibial articulation line was considered a “varus” if the angle was negative.

#### Tibiotalar joint rotation angle

**Fig 9:**
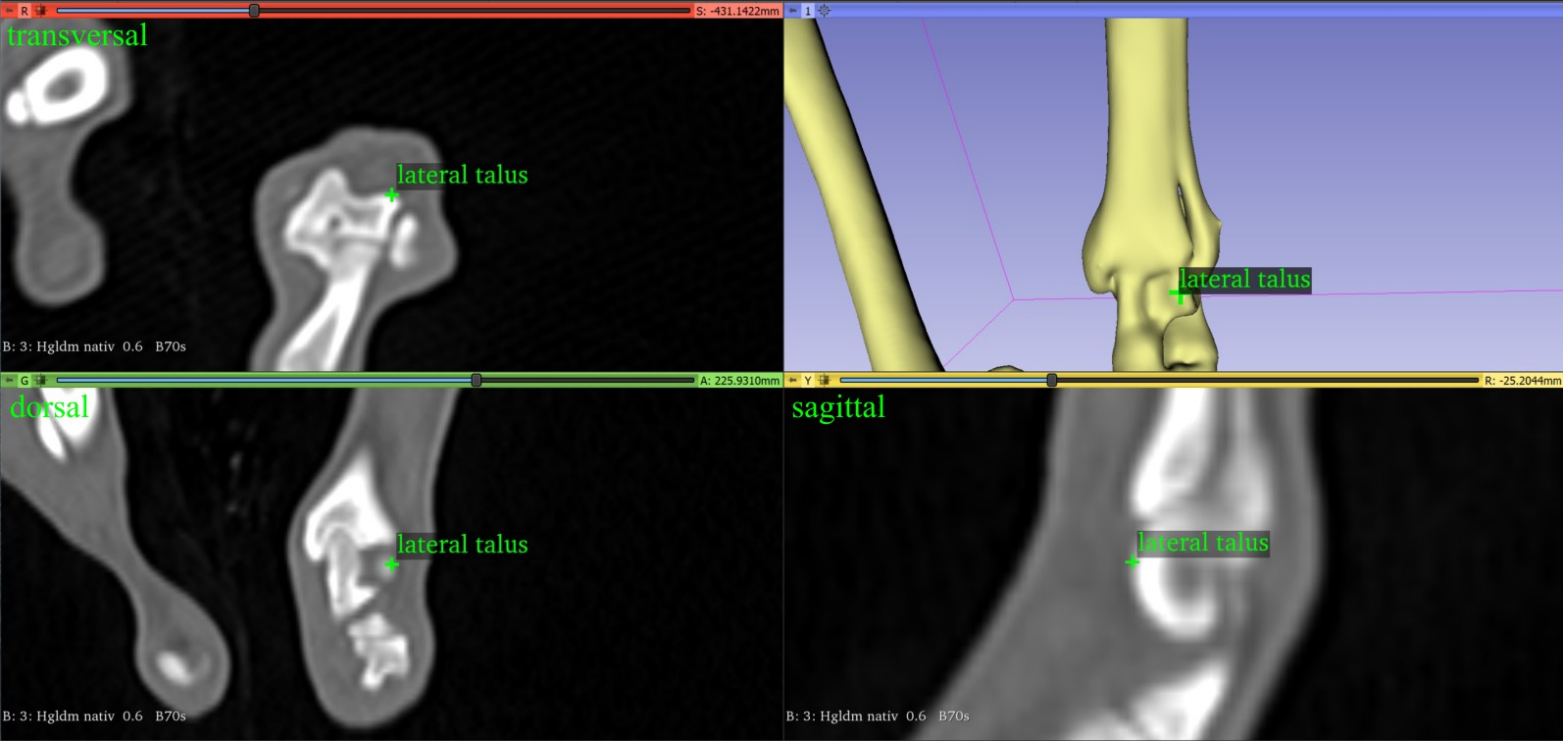
The most cranial point on the lateral part of the articular surface of the trochlea of the talus of the left tibia of a dog.

We calculated the tibiotalar rotation angle using two vectors: the talus front line, defined as the line between the most cranial point on the lateral and medial articular surface of the trochlea of the talus (Fig 9), and the distal tibial front line [13, 14]. Both vectors were projected into the transverse plane defined by the longitudinal axis of the tibia as the normal vector of the transverse plane. If the talus rotated laterally towards the tibia, we termed the rotation as external rotation, and if the talus rotated medially towards the tibia, we termed the rotation as internal rotation. In the case of an external rotation, the angle was defined as being negative. In the case of internal rotation, the angle was defined as positive.

### Evaluation of the method

#### Application and feasibility in clinical CT data

To test the feasibility of the developed 3D Slicer extension software, we retrieved computed tomographic studies of 113 pelvic limbs of dogs that underwent CT-examinations for various clinical reasons unrelated to this project, from the picture archiving and communication system (dicomPACS, Oehm & Rehbein, Rostock, Germany) of the Clinic of Small Animal Surgery and Reproduction, Center of Veterinary Clinical Medicine, LMU Munich. None of the dogs had previous surgery, or the presence of a bone neoplasia on the limb used, which were the only exclusion criteria. Inclusion criteria were the presence of images of the entire hind limb (from hip to mid-metatarsal). Therefore, both orthopedically healthy and unhealthy dogs were included. TDogs were positioned similar to a ventrodorsal or dorsoventral pelvic radiograph for canine hip dysplasia screening, with the coxofemoral, stifle and tarsal joints extended during scanning, but perfect symmetry was not required. Based on routine clinical protocols, CT-scans were performed with a helical multi-slice CT scanner with a fixed detector array design (Somatom Definition AS VA48A_02_P12, 64 Excel Ed. software Somaris/7 syngo CT VA48A Siemens Healthcare GmbH, Erlangen, Germany). All CT-data sets were acquired in helical mode with a detector slice thickness set to 0.6 mm, tube voltage set to 120 kV, tube rotation time set to 0.5 – 1 s, pitch set to 0.8–1 and tube currents variably adjusted according to patient size. The reconstructed slice thickness and increment were identical and ranged from 0.6 mm to 0.75 mm, resulting in gap-free image stacks and thus continuous three-dimensional CT-data. Images were reconstructed using a bone algorithm (deconvolution filter: kernel 60 or 70). We exported the DICOM images and imported them into 3D Slicer using the self-programmed extension software plug-in. After importing the CT data into the program, the bones were segmented by thresholding. A pop-up window displays all available measurements, along with the points required for each measurement and a written description and example images of each reference point. The operator selects and sets reference points using triplanar orthographic MPR and three-dimensional volume rendering CT images, as described above. We used a standard bone window (500 HU window center, 2500 HU window width) and VR thresholds were set at 300 HU for the bone segmentation. Once the reference points have been set in a CT study, the measurements of the above angles, as well as the three-dimensional Cartesian coordinates of each reference point, can be read and exported to a file. In addition to using a graphical interface based on MPR and VR-CT images to visually select reference points to determine their three-dimensional coordinates, three-dimensional coordinate values can also be entered numerically into the program.

#### Validation by comparison with reference standard

To evaluate and validate our program, we compared the angule measurements of our 3D Slicer extension software with the commercially available three-dimensional medical imaging software, VoXim® (version 6.5.1.1 (T2160910) Copyright©) from the medical imaging company IVS Technology GmbH [LLC], Chemnitz, Germany, which had full official medical device approval [59, 60]. VoXim® included routine clinical MPR, VR and segmentation functions. It was designed for three-dimensional angular measurements and voxel imaging based on the DICOM-image data-based coordinate system. VoXim® has been validated and used in clinical and anatomical studies [58, 59] and was therefore used as a reference standard for comparison [61]. Based on anatomical reference points and axes, additional anatomically oriented three-dimensional coordinate systems could be introduced into VoXim®, allowing three-dimensional angular measurements [61]. We used the same predefined projection planes and the same reference points for our 3D Slicer extension software as in VoXim® [61].

CT-data of 113 pelvic limbs were imported into VoXim®, the anatomical reference points were set as described above and we calculated femoral torsion, femoral varus (or valgus), femorotibial rotation, tibial torsion, tibial varus (or valgus) and tibiotalar rotation angles. The angular measurements and angular orientation of each hindlimb were manually read and exported. The three-dimensional coordinates of each reference point were exported from VoXim® and imported into our 3D Slicer extension, in order to repeat the angle measurements and export the angle measurements, including the angle orientation.

#### Statistical analysis

The circular mean and circular standard deviation were calculated for the difference between the two methods for each angle. Modified Bland-Altmann plots using a Von Mises distribution were created to compare angle measurements from the 3D Slicer extension and VoXim®.

## Results

CT-data of canine hind limbs could be imported, opened, and viewed in 3D Slicer and its extension software, the operator could set the reference points based on the described anatomical localization, fit spheres, perform all angular measurements and read out the results including the automatically determined angle direction. The external coordinates of the anatomical reference points based on the measurements that were performed with the reference standard VoXim® in CT-scans of 113 canine pelvic limbs could be imported and the angular measurements including the angle directions could be calculated, read out and exported. Therefore, we believe that the scheme is feasible.

**Fig 10:**
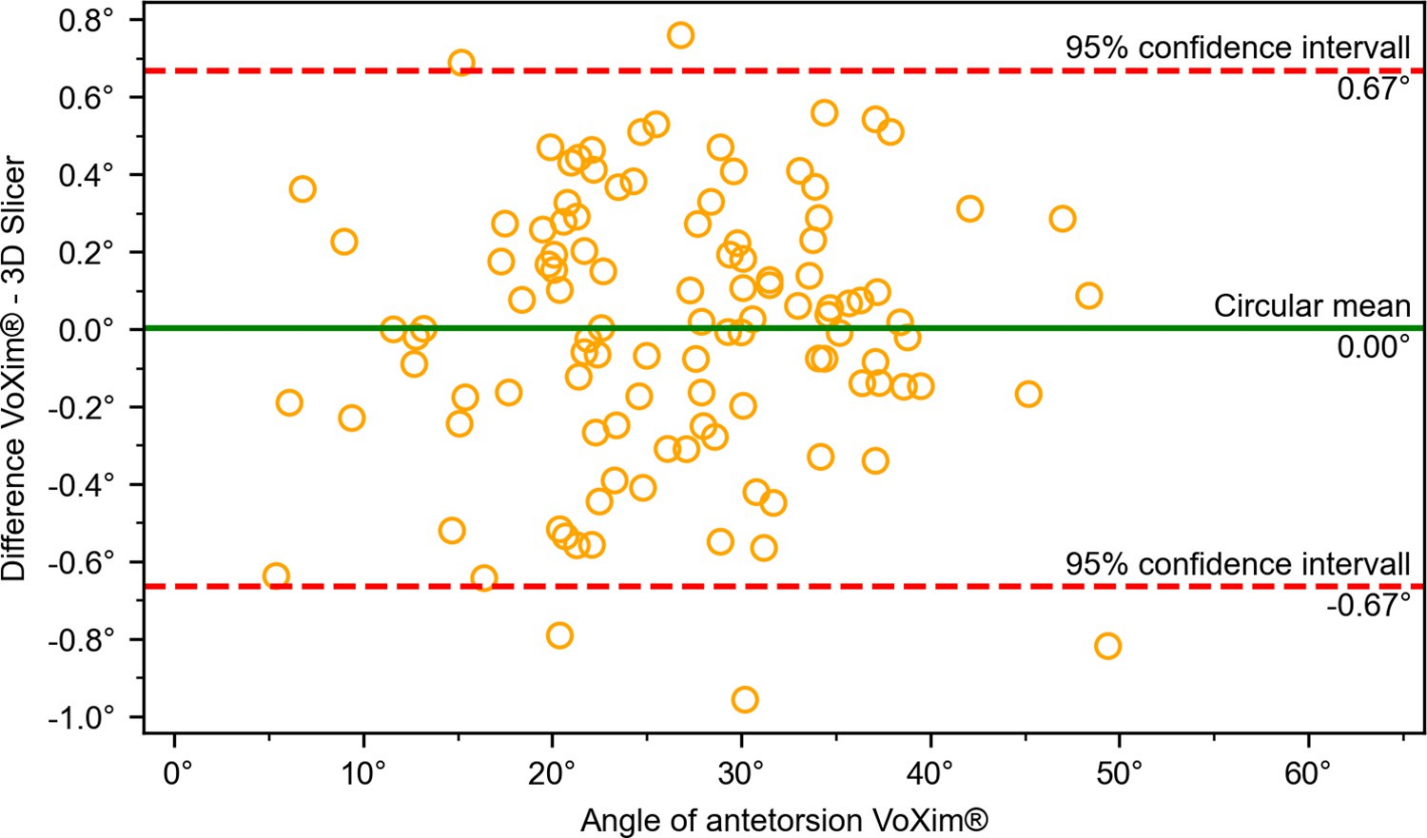
Comparison of the VoXim® method with the 3D Slicer method using a modified Bland-Altmann-Diagram for the antetorsion angle measurement.

**Fig 11:**
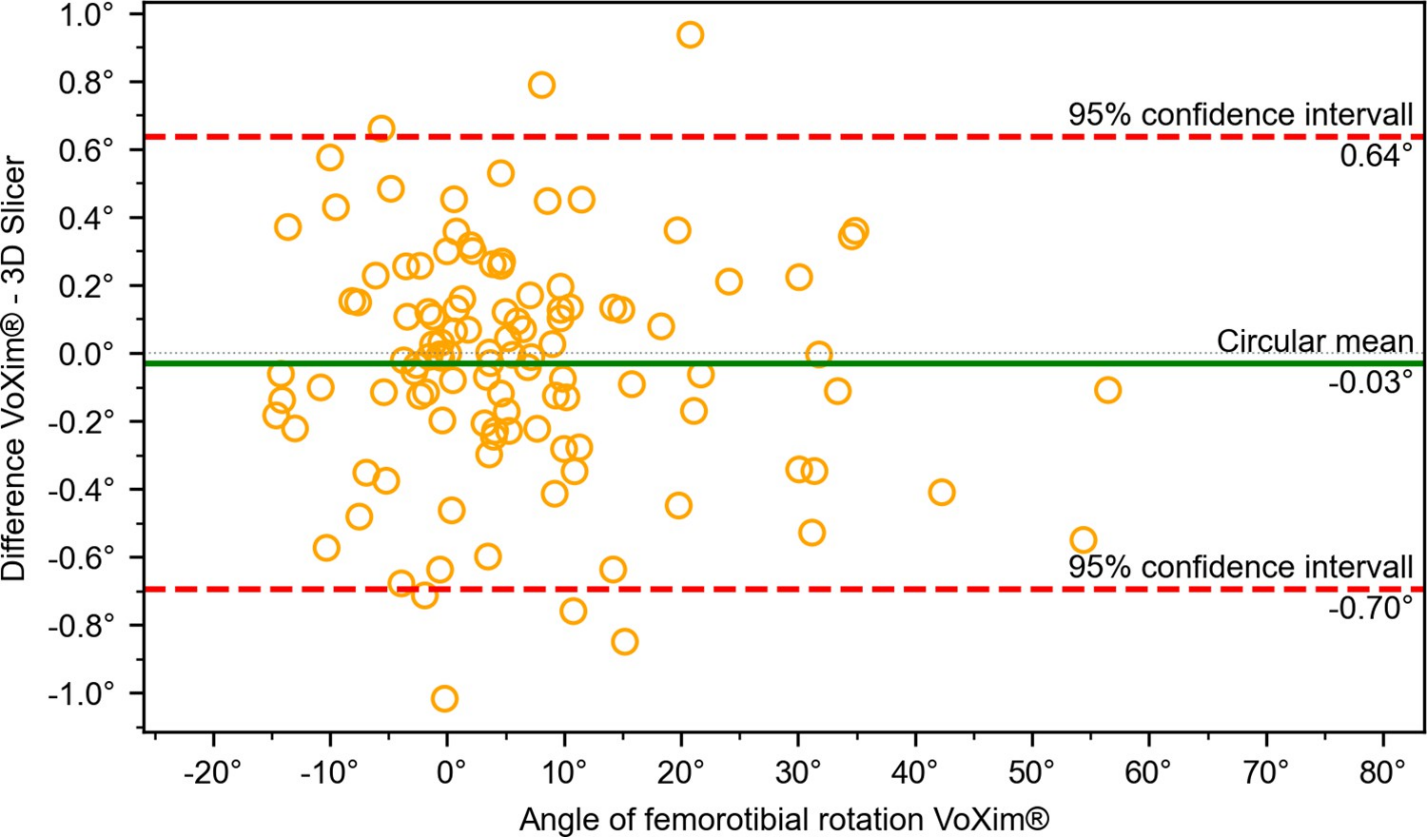
Comparison of the VoXim® method with the 3D Slicer method using a modified Bland-Altmann-Diagram for the femorotiabal rotation angle.

**Fig 12:**
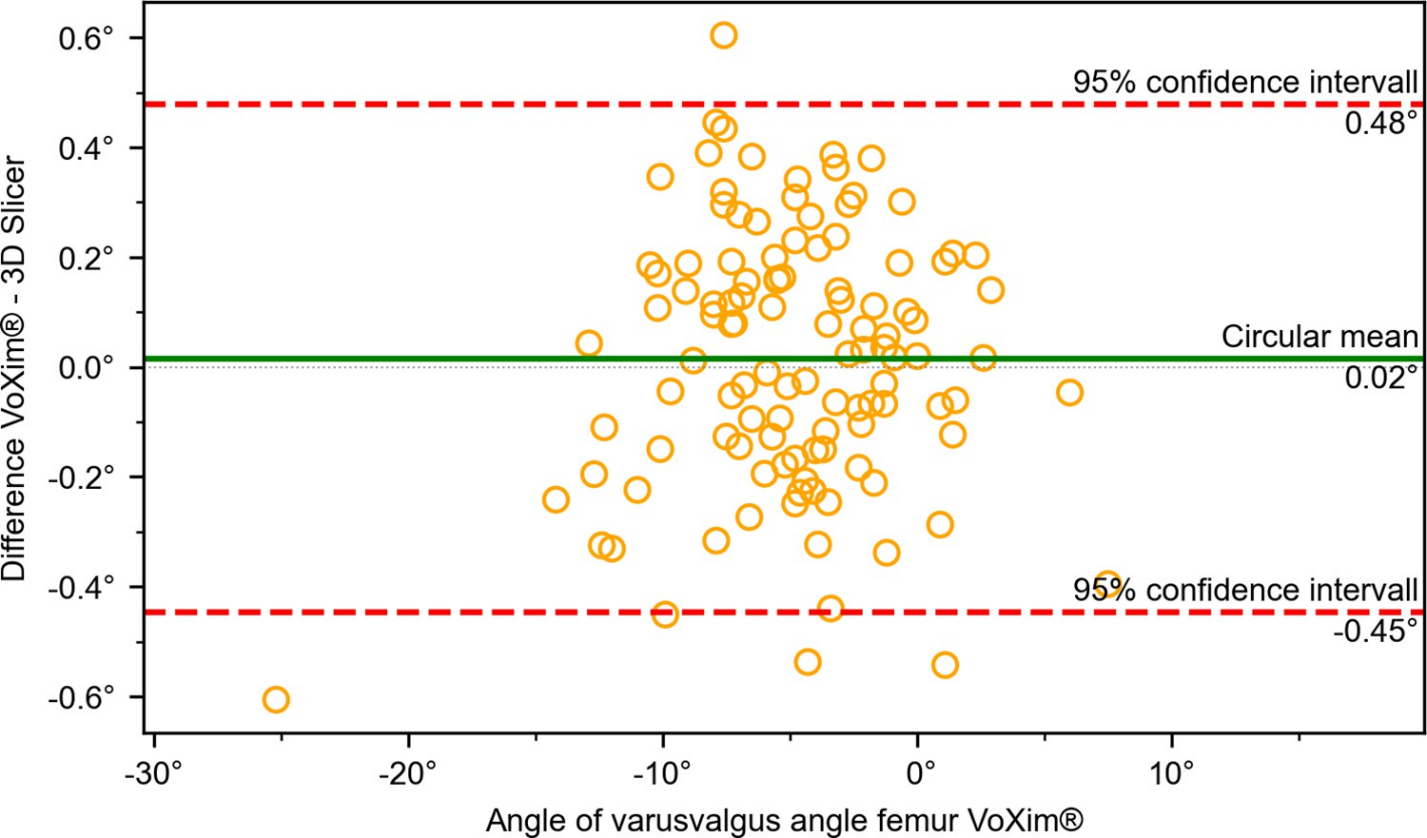
Comparison of the VoXim® method with the 3D Slicer method using a modified Bland-Altmann-Diagram for the femoral varus/vagus angle.

**Fig 13:**
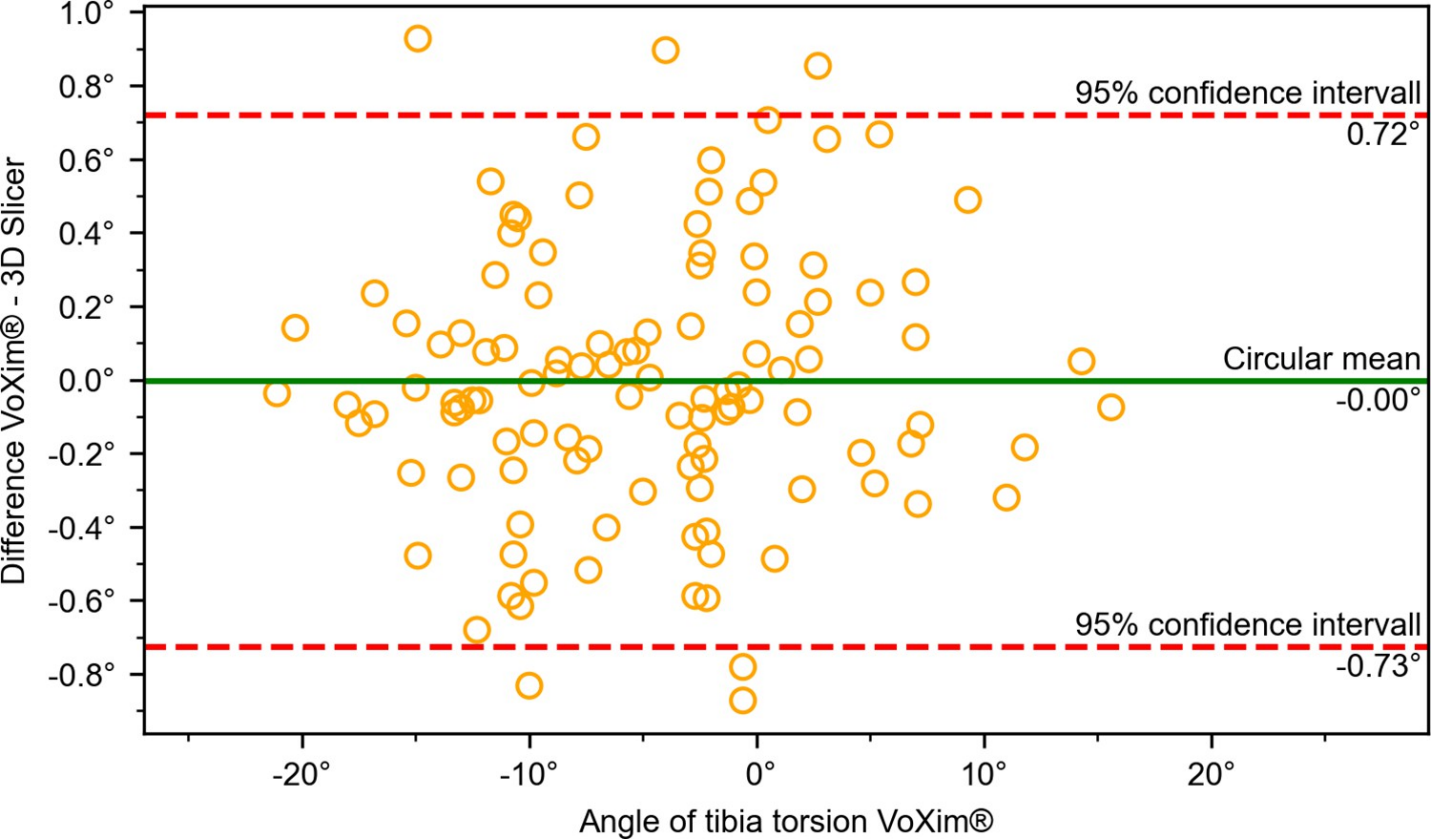
Comparison of the VoXim® method with the 3D Slicer method using a modified Bland-Altmann-Diagram for the tibial torsion angle.

**Fig 14:**
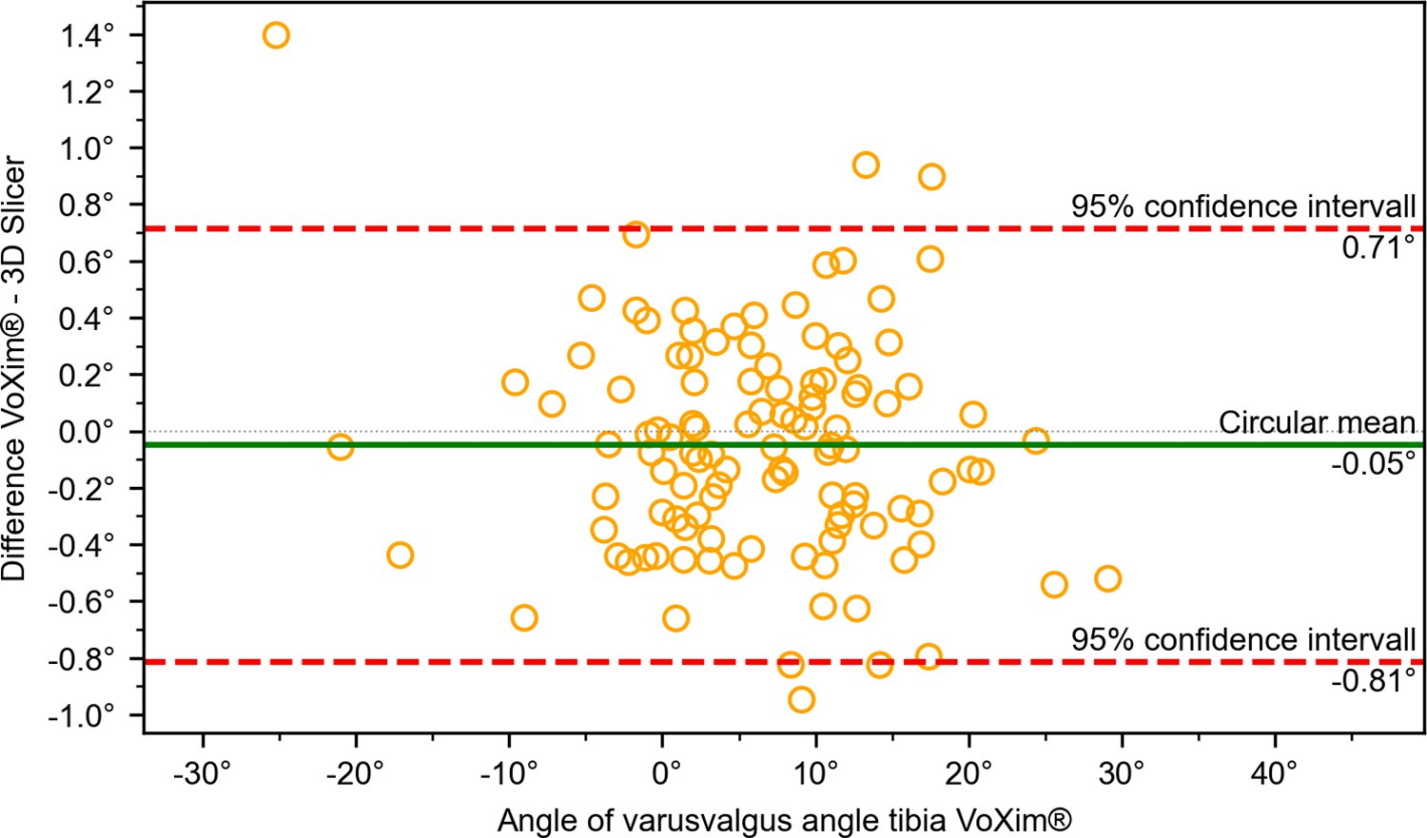
Comparison of the VoXim® method with the 3D Slicer method using a modified Bland-Altmann-Diagram for the varus/valgus angle of the tibia.

**Fig 15:**
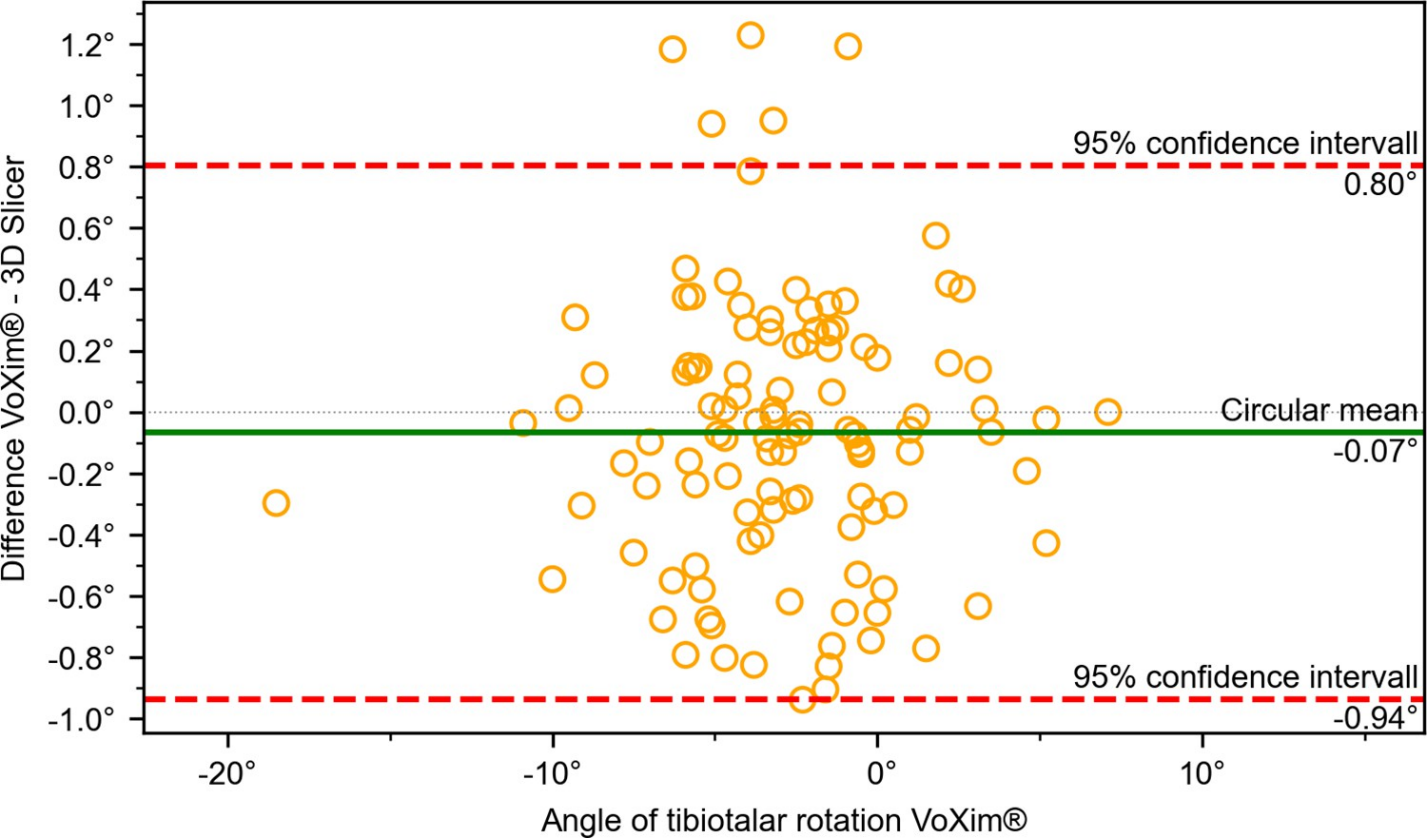
Comparison of the VoXim® method with the 3D Slicer method using a modified Bland-Altmann-Diagram for the tibiotalar rotation angle.

The results of the comparison between our 3D Slicer extension and the VoXim® program showed no significant differences between the two methods (Figs 10–15) (Table 1). The Bland-Altman plot did not show any systematic differences or significant outliers between the angle measurements of the two methods. The circular mean showed no significant deviation, with all comparisons below 0.07° and 0.00 (Figs 10–15). Based on these results, we believe that the angle measurements made with the 3D Slicer extension are accurate.

**Table 1:** Circular mean and circular standard deviation of the difference between the VoXim® method with the 3D Slicer method for each angle in degrees.

Index	Tibial torsion (°)	Varus/ valgus tibia (°)	Varus/valgus femur (°)	Tibiotalar rotation (°)	Femorotibial rotation (°)	Antetorsion (°)
**Circular mean**	-0.00	-0,05	0,02	-0,07	-0,03	0,00
**Circular standard deviation**	0.37	0.39	0.24	0.44	0.34	0.34

## Discussion

We developed a 3D Slicer extension software that allowed CT-based measurements of various clinically relevant bone and joint angles of the canine hind limb within an entire three-dimensional CT-data set using the coordinates of reference points and three-dimensional mathematical definitions of projection planes based on vector calculations rather than measurements in two-dimensional transverse, MIP-, MPR- or VR-images. Angular measurements using a primary three-dimensional approach are described for the femur based on three-dimensional mesh models and computer-aided design-based software using optical scans of normal canine cadaver bones [47]. This technique allows automatic measurement of multiple morphological parameters in normal femurs, which may be much faster than our method because we manually set reference points, which requires manual operations and is very time-consuming. Fast and fully automated angle measurement is a long-term goal, but its successful application in routine patient studies and deformed bones needs to be demonstrated. To show the feasibility of our program, we used clinical CT-data that contained many heterogeneous scans of dogs of different sizes and disorders, including presumed normal bones and hind limbs, as well as cases of patella dislocation and severe bone deformities, but which may under-represented cases of severe complex deformities. Even if our technique proves to be feasible in clinical scans, it will still need to demonstrate its prospective clinical utility and value in severely deformed skeletal patients undergoing orthopedic surgery. By calculating the normal vector, we mathematically define the projection plane for angle measurements to minimize the variation of the measurement plane based on visual judgments. To standardize the projection planes of torsion angle measurements in three-dimensional volume rendering views of cadaveric canine tibia CT- scans, other researchers used superimposition of proximal and distal reference points of the mechanical axis [60]. In contrast, we used a three-dimensional RAS (Right, Anterior, Superior) patient Cartesian coordinate system of 3D Slicer to read the three-dimensional information of the CT-data and measured angles in this coordinate system using vector calculations and vector projection in planes. Vector calculations are advantageous for our purposes because they are independent of the reference frame. One coordinate system can be mapped to another coordinate system, and three-dimensional coordinates can be converted between different three-dimensional coordinate systems [55]. We validated the accuracy of our program by comparison with another software, which is a limitation compared to anatomical comparisons of anatomical bones, which can be considered the true gold standard. VoXim® is designed for three-dimensional angle measurements, is approved for medical devices and validated in other studies, allowing us to use and compare data from many clinical scans [58, 59, 61]. CT images of normal bones alone are also of limited value, and three-dimensional macroanatomical measurements of cadaveric bone are also difficult to perform and may not necessarily be more accurate. Measurements in repeated CT-scans of the same hind limbs in different positions and repeated measurements by the same and a different observer were not performed for the 3D Slicer extension used in this project but were previously evaluated using the same reference points and the VoXim® software [61, 62].

Using the same coordinates, there were small differences between the 3D Slicer extension and the VoXim® software. When transferring coordinates from VoXim® to the 3D Slicer extension software, two decimal places were used, which may have been rounded before export, and more decimal places were used in the software’s internal calculations. This effect may explain the small differences between the two procedures. Regardless of the cause, these small differences are likely to be considered clinically irrelevant, based on current surgical techniques. This may change in the future, with the useof computer- and robot-assisted surgeriy.

Given the accuracy of the mathematical vector calculations, the inter-observer agreement in the reference point selection process is likely to be the weakest link. Therefore, improving the accuracy and precision of the measurement process means improving the reference point selection process. New reference points and better determination of existing reference points using mathematically supported semi-automatic or automatic tools could be two main potential future strategies. Furthermore, it could be the starting point for a fully automated workflow that places the reference points using deep-learning methods [63]. As a form of artificial intelligence (AI), deep learning methods require a large number of labelled training samples, which are tedious to generate manually. Therefore, we have tried to design an intuitive and simple user interface of the program that is as easy and fast to use as possible. In order to make the technology available to other interested veterinary clinics and research institutions for collaborative use and further development, this study has been implemented using the open-source 3D Slicer and our extension software tool has also been publicly released along with the source-code. For the future, this plug-in can be easily extended to include and implement additional new or alternative reference points and angle measurements.

In this first version we included femoral torsion, femoral varus (or valgus), femorotibial rotation, tibial torsion, tibial varus (or valgus) and tibiotalar rotation angles, as these are of clinical relevance and are commonly reported in the veterinary orthopaedic literature. To date, there are limited descriptions of joint rotation angle measurements on CT. We included these measurements because of their relevance to the canine stifle joint with patellar luxation. Changes in bony torsion angles may be associated with, caused by or compensated for by joint rotation angles, but further research is needed to determine their clinical relevance and physiological variation based on a different patient positions, as canine stifles should be rotationally stable in extended position and allow slight rotation in flexed position. Bone torsion deformity and joint rotation may be superimposed at several bone and joint levels and may cancel each other out across the limb. Similarly, varus and valgus angles of the femur and tibia might be compensated by each other. Therefore, a thorough and comprehensive understanding of the entire canine hindlimb alignment is required for a targeted and successful treatment. In this project, we addressed torsional bone and rotational joint deformities in the transverse plane of the limb, as well as medial or lateral bony deviation expressed as varus or valgus angles in the dorsal plane, but not pro- and recurvatum deformities in the sagittal plane and our method also lacks three-dimensional angular measurements of the coxofemoral joint and paws.

Different modalities and multiple methods based on different reference points can be used to measure canine hindlimb angles, with computed tomography considered to be more accurate and reproducible than radiography [39]. Reference values are considered to be specific to the technique used and cannot be easily compared or transferred between different modalities and measurement techniques within the same modality, as has been shown in human medicine [64]. The same may be true in veterinary medicine, and reference values for dogs may also be breed-specific. This fact may explain the differences in angle measurements observed by different authors [1].

Descriptions of reference points and projection planes based on two-dimensional images lack the third dimension, leaving room for variation, so we described reference points in all planes.

In the VR-views of our current program there is lack of visibility of reference points within or behind bones compared to the VoXim® reference software, where reference points are visible within semi-transparent bones or virtual overlays of reference points are available. The extent to which these technical differences may a favorable, neutral or unfavorable effect on the selection of reference points and thus on the overall precision of the technique is currently unknown and difficult to assess. The use of a fixed orthogonal MPR-view is a technical limitation. The use of a completely free MPR tool that supports any plane might improve or at least simplify the reference point setting in scans where the dog is placed obliquely in the scanner.

We set the femoral head center using the center of a three-dimensional fitted sphere, which is consistent with recent work [12, 39], rather than using the center of a circle in a two-dimensional image. Our best fitting sphere based on the least-square-method, requires an operator to set five reference points along the femoral head, which are not specified individually, but should be distributed on the subchondral bone of the articular bearing area rather than in the fovea capitis or along the femoral neck. We assumed that the canine femoral head was a true sphere, although, human femoral heads appear to be better represented by superovoid fitting than by a true sphere [65], but even if this were similar in the dog, we would probably consider this a minor error and drawback of our method. To define the femoral head-neck axis with a second reference point, we used the femoral neck base center instead of a femoral neck center, which was originally described for radiography [37] and can also be used alternatively in CT [40, 42, 48]. The description of how to find the femoral neck center in CT-images was less clear than the description of the femoral neck base center [39] and was also more difficult to identify and define anatomically [62]. This, and the fact that two alternatives are described, without a final consensus on which is more accurate and precise, is in our opinion a weakness of the current approach that could benefit from further improvements. Currently, our reference points for femoral torsion and varus angles on the femoral condyles are consistent with the literature, but in future versions of the program their anatomical accuracy and precision may benefit from additional features such as fitting spheres or centroids as described for femoral CAD-meshes based on optical scans of cadaveric bones [47]. The same applies to the subchondral surface center of the tibial condyle for tibial varus angle measurement. Our reference point for the tibial torsion measurement is consistent with previous CT-based measurements. However, we believe that our extended description and use of the reference points in true three-dimensionality and our three-dimensional angular vector calculations are an improvement over measurements in two-dimensional images, but further clinical studies are needed to prove this assumption. As with a routine diagnostic evaluation of a clinical imaging study, angle measurements can be performed using the onboard viewing software of the CT-scanner or additional external workstations with additional medical image viewing software, such as the 3D Slicer extension program of this project. The 3D Slicer is not approved for clinical use in human medicine based on medical device regulations (“not an FDA-cleared product”) [56], and nor is our 3D Slicer extension program, which is designed for veterinary medicine. The intended use of 3D Slicer and its extensions and clinical research applications and these experimental tools cannot be packaged within the “FDA-cleared” workstations used clinically [56]. DICOM images must be transferred from the CT scanner to a separate workstation which is a disadvantage in a clinical setting. Alternatively, if three-dimensional coordinates could be read out at the CT-scanner workstation, reference points could also be set at the CT-scanner and their coordinates entered directly into our 3D Slicer extension program, to calculate the angles, avoiding the need to transfer CT-studies.

Currently, in many countries, the use of DICOM-viewer software for diagnostic imaging in animals for veterinary applications does not, to our knowledge, require regulatory medical device approval or legal licensing, including hardware such as computer and monitors used for diagnostic purposes [66]. Medical imaging equipment and specialized viewer software for three-dimensional applications can be expensive. Financial limitations of pet owners, commonly without health insurance for their animals, often play a role in veterinary medicine and free open-source software may provide a cost-effective alternative and opportunity for veterinarians to diagnose and treat canine patients in small animal practice [66].

Human studies have described the (temporary) reduction of fractures using a three-dimensional printed template for an external fixator in polytraumatic patients unable to undergo anaesthesia [67, 68]. This could also be used in veterinary medicine, as well as other pre-operative surgical planning using three-dimensional printing. Open source software such as ours could help research in this direction, as complicated fractures as well as severe and complex angular hind limb deformities in dogs require precise morphological evaluation using diagnostic imaging to ensure successful orthopedic surgery. With our open-source 3D Slicer extension software we are providing a freely available tool for veterinary orthopedic surgeons. We hope to improve angle measurements in CT-scans of canine hind limb deformities by providing true three-dimensionality.

## Supporting information

S1 Data (XLSX)

S2 Data (ZIP)
